# Bacterial community response to novel and repeated disturbances

**DOI:** 10.1111/1758-2229.70022

**Published:** 2024-10-10

**Authors:** Susannah Halbrook, William Wilber, Mary Elizabeth Barrow, Emily C. Farrer

**Affiliations:** ^1^ Department of Ecology and Evolutionary Biology Tulane University New Orleans Louisiana USA; ^2^ Department of Biological Sciences University of Notre Dame Notre Dame Indiana USA

## Abstract

Disturbance response and recovery are increasingly important in microbial ecology, as microbes may recover from disturbances differently than macro communities. Past disturbances can alter microbial community structure and their response to subsequent disturbance events, but it remains unclear if the same recovery patterns persist after long‐term exposure to stress. Here, we compare bacterial community composition in a community that experienced 2 years of monthly salinity addition disturbances with a community that has not experienced salinity additions. We then track the response and recovery to an additional salinity addition based on past disturbance exposure. We tested the following hypotheses: first, communities with a repeated disturbance history will have a different community composition than communities without a disturbance history; second, communities exposed to repeated disturbances will undergo a different recovery trajectory than communities experiencing a novel disturbance. We find that repeated disturbances alter community composition and affect community response and recovery to a subsequent disturbance after 2 years, primarily through increased resistance. This work enhances our understanding of microbial temporal dynamics and suggests that novel disturbances may pose a threat to microbial community structure and function.

## Graphical abstract

In this disturbance experiment, we compared the resistance and resilience of soil bacterial communities with and without a history of disturbance to a subsequent salinity disturbance. We find that past salinity disturbances alter community composition and increased community resistance to future disturbances.

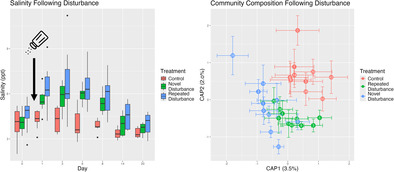

## INTRODUCTION

The effects of disturbance history on community structure and stability have been well studied in animal and plant systems, but only recently has been studied in microbes (Shade et al., 2012, Bardgett & Caruso, 2020; Philippot et al., 2021). The distinct physiologies and life histories of microbes compared to macro‐organisms may lead to microbes exhibiting unique response patterns to environmental disturbance, making it necessary to re‐examine these questions in microbial systems. For example, the high diversity and functional redundancy of microbial communities (Chen et al., 2022; Fierer, 2017), paired with short turnover times (Gibson et al., 2018; Powell, 1956) and ability to use dormancy to survive inhospitable periods (Blazewicz et al., 2014; Lennon & Jones, 2011) could lead to distinct community disturbance responses compared to animals and plants.

The disturbance regime of an ecosystem can impact microbial composition by repeatedly selecting for microbial taxa that are tolerant of, or can recover from, disturbance stressors over long time periods. Disturbance experiments have found that past disturbances alter microbial composition (Berga et al., 2012; Santos‐Medellín et al., 2017) and function (Bérard et al., 2012; Bouskill et al., 2013; Kaisermann et al., 2017; Meisner et al., 2015) following subsequent disturbances compared to naïve communities, but examples of the effects of long‐term disturbance regimes on community structure (Nielsen & Ball, 2015) are less common. Theoretical work shows that a history of environmental variation affects the functioning of microbial communities (Hawkes & Keitt, 2015), and the field and laboratory experiments that have tested long‐term, repeated disturbances have also shown that they alter diversity and composition (Osburn et al., 2019; Preece et al., 2019; Seitz et al., 2022; Shen et al., 2016), functional diversity (Seitz et al., 2022), function (Canarini et al., 2021; Evans & Wallenstein, 2012, 2014; Fuchslueger et al., 2016), and network structure (Osburn et al., 2019) in a diverse array of systems and stressors.

Historic disturbance regimes may not only affect community structure and function but may also affect the community's recovery to future disturbances. Repeated disturbance may increase a community's resistance and resilience as the community adapts to the recurring environmental stress, where resistance describes the degree of compositional change following a disturbance and resilience describes how quickly the community returns to its pre‐disturbance composition (Shade et al., 2012). Considerable research effort has examined the effects of drought stress on soil microbiomes and finds that past drought events, whether over short or long‐term periods, leads to increased resistance (Bouskill et al., 2013; Canarini et al., 2021) and/or resilience (Bérard et al., 2012; de Nijs et al., 2019) to future drought stress. Drought‐stressed communities have also been found to adapt to drought by altering their recovery strategy (Evans & Wallenstein, 2014). However, microbial response to other types of disturbances, like salinity, fire, and heat shock, have yielded less consistent results, including finding little or no community resilience (Berga et al., 2012; Bernhard et al., 2015, Calderón et al., 2018; Feckler et al., 2018; Hu et al., 2018; Jurburg, Nunes, Stegen, et al., 2017; Shen et al., 2016). A more thorough investigation of microbial responses to other disturbances, like has been done with drought stress, would lead to more a conclusive understanding of the effect of historic disturbance regimes on microbial community recovery and adaptation.

We tested the effect of repeated disturbances on soil bacterial community structure and recovery in a brackish marsh in SE Louisiana using salinity pulses as the disturbance. Coastal wetlands are an understudied habitat (Carini et al., 2016) prone to frequent and rapid changes in salinity and predicted to experience increased mean salinity over time with sea level rise (Fagherazzi et al., 2019). The frequent abiotic fluctuations and long‐term salinity changes provide a useful context to examine how soil communities respond to salinity stress follow a long‐term disturbance regime. The few studies that have tested salinity stress find communities to have inconsistent recovery and that the frequency of the disturbance impacts community composition (Berga et al., 2012; Hu et al., 2018; Mobilian et al., 2020).

We work to expand our understanding of microbial response to salt stress in natural environments by implementing a field‐based disturbance experiment, using a two‐year monthly salinity addition regime as the historic disturbance. We assessed differences in community composition between communities with no artificial disturbance versus 2 years of repeated disturbance history. We then compared the recovery trajectory of bacterial communities to an additional salinity disturbance in the community with the repeated disturbance history versus the community for which the salinity addition was a novel disturbance. First, we hypothesize that the community with the repeated disturbance history will have different community composition than the community without a disturbance history, indicating the effect of long‐term, repeated disturbances on composition. Second, we hypothesize that the community exposed to repeated disturbances will undergo a different recovery trajectory than the community experiencing a novel disturbance. Specifically, we predict that repeated disturbances will lead to less rapid and less extreme compositional change following the salinity addition (increased resistance), and a quicker recovery to the initial community composition (increased resilience) compared to novel disturbance community.

## EXPERIMENTAL PROCEDURES

### Experimental design

In the fall 2018, 24 permanent 1 × 1 m plots were established in the Pearl River WMA, LA (30°14′14.9″N 89°37′25.6″W). Plots were organized into three treatments: repeated disturbance (2‐year monthly disturbance), novel disturbance (single disturbance event), and control (no disturbance), where each treatment had eight plots. Plots were organized in a block design, where each block contained one plot from each treatment for a total of eight blocks. Each plot was 5–10 m from neighbouring plots, and all plots represent a native marsh plant community, dominated by *Spartina patens*. Repeated disturbance plots received a monthly addition of 750 g of salt (Instant Ocean Sea Salt, Blacksburg, VA) (Moon & Stiling, 2002) for 2 years, increasing salinity by about 33% but returning to initial levels within a month, to establish a 2‐year repeated disturbance regime.

In December 2020, soil samples were collected from all plots (Day 0, “pre‐treatment”) before adding 750 g of salt to the repeated disturbance and novel disturbance plots as the subsequent disturbance event. An unexpected rain event on Day 0 following the sample collection and salinity addition diluted and washed away the salt so that there was no increase in salinity on the following day. To account for this, salt was added again the following day, this time successfully increasing salinity within 24 h. Day 0 refers to pretreatment conditions (before any salt was added), and Day 1 (and beyond) refers to 1 day after the second salt addition that successfully increased salinity. Following the salinity addition (Day 1 and beyond), samples were collected in the following time sequence: every other day for the first week, once per week through the first month, and every other week for a second month. A total of 10 time points were sampled, including Day 0, which will be referred to as the number of days post‐disturbance (ranging from Days 1 to 55).

### Sample collection

Each collection day, samples were collected from a randomly selected, non‐repeating subplot within the plot (excluding the outer 20 cm of the plot to avoid edge effects). Soil pore‐water salinity was measured at 15 cm depth using sippers to suction up pore water and dispense into a falcon tube before measuring with a salinity meter. Daily salinity was measured at two locations in each plot, the plot centre, and the daily subplot, to capture spatial heterogeneity. These values were averaged for statistical analyses. Once pore water was collected, soil samples were taken within the subplot with a sterile soil corer to 10 cm depth. Soils were kept on ice until returning to the lab.

### Molecular methods

Upon returning to the lab, samples were homogenized then treated with PMAxx (Biotium Inc., Freemont, CA) to remove relic DNA (free‐floating, extracellular DNA or DNA in dead cells). PMAxx is a photo‐sensitive reagent that binds to free‐floating DNA and prevents downstream amplification. The result is the amplification only of DNA from intact, living cells. Relic DNA has been found to represent about 40% of amplified prokaryotic DNA in soil samples (Carini et al., 2016; Lennon et al., 2018), so removing it provides a more accurate picture of the live bacterial community, which is important given the rapid time sequence of the experiment. Briefly, 0.3 g of soil was suspended in 3 mL of PBS buffer and 7.5uL of PMA to reach a final sample concentration of 50 mM PMAxx. Samples were incubated in the dark for 10 minutes followed by a 15‐minute light exposure on ice with a 500 W Halogen bulb at a distance of 20 cm to activate the PMAxx (Ramírez et al., 2018). Samples were inverted and/or rotated to mix once per minute during the dark and light incubation. Samples were then stored at −20°C.

DNA was extracted with the Qiagen PowerSoil Kit following the manufacturer's protocol, with the exception that a slurry of 960 μL of soil from the PMAxx protocol was added instead of dry soil (Carini et al., 2016). Samples were standardized to 2 ng/μL before dual‐step PCR, done in duplicate, to amplify the 16S region with primers 515F/806R (Farrer et al., 2021). PCR product was pooled, purified and concentrated with AMPure, and sequenced on Illumina Miseq v3 (300 bp PE) at Duke Sequencing Core, Duke University, Durham, NC.

### Bioinformatics

Sequencing data was processed with an ASV method using the Qiime2 (Bolyen et al., 2019) and DADA2 (Callahan et al., 2016) bioinformatic pipelines. Reads were first trimmed where quality scores dropped below ~30, then quality filtered, denoised, and paired reads were joined. Potential contaminants identified from six control samples were removed using the R package decontam (prevalence option) (Davis et al., 2018). The resulting data were rarefied to 5500 reads per sample for dissimilarity analysis, singletons were removed from the rarefied data for compositional analysis, and unrarefied with relative abundance was used for taxonomic analysis. Taxonomy was assigned using Greengenes (DeSantis et al., 2006).

### Statistical analysis

To assess how the salinity addition increased plot salinity, we used linear mixed effects models to test the effect of Treatment (control, repeated disturbance, novel disturbance) on salinity on each day of the experiment using the function lme() with Plot and Block as nested random effects in the R package nlme (Pinheiro et al., 2023). ANOVAs tested significance, and post‐hoc tests with the function glht() from the R package multcomp (Hothorn et al., 2008) compared the salinity levels between treatments on each day to confirm the two salt treatments (repeated disturbance and novel disturbance) did not differ from each other.

To test the first hypothesis and compare the pre‐treatment communities, the data were subset to only include the Day 0 samples. A PERMANOVA using adonis2() in the R package vegan (Oksanen et al., 2022) was used to test the effect of Treatment on composition using the strata argument to restrict permutations by block. Dispersion was calculated with the function betadisper(). Subsequent pairwise PERMANOVAs were used to compare Day 0 composition between each treatment by further subsetting the Day 0 dataframe to only include two treatments per comparison. A dbRDA ordination plot was used to visualize the Day 0 communities with the capscale() function in vegan, conditioned on block. The points were plotted by extracting the CAP scores from the capscale() output and plotting in ggplot2 (Wickham, 2016).

To test the second hypothesis, that the treatments had different recovery trajectories, PERMANOVAs were used to test the effect of Treatment, Day (as a factor), and their interaction on community composition over the whole collection period. In order to account for repeated measures of plots over time, PERMANOVAs were done manually in R with different types of models and randomization restrictions (Simpson, 2020) using adonis2() and the how() function in the package permute (Simpson, 2022). First, to calculate the correct *F*‐statistic for the effect of Treatment, we ran an adonis2() model testing the effect of Plot + Treatment and extracted the sums of squares for the Treatment variable (divided by df) and divided it by the sums of squares for the Plot variable (divided by df); this accounts for the fact that in a repeated measures design, the denominator in the *F*‐statistic is the whole‐plot error rather than the residual error (Simpson, 2020). We then performed a permutation test with 999 permutations, randomizing the plots freely within blocks (comparing Treatments), but not randomizing within plots (individual samples), using the how() function. For each permutation, we ran the same adonis2() model and calculated the *F*‐statistic for the Treatment effect. We then calculated a *p*‐value by comparing the *F*‐statistic of our actual data to the distribution of *F*‐statistics of the randomized data. To test the effect of Day, we fit an adonis2() model testing the effect of Plot + Day and restricted permutations within plot, which compares samples taken over time to only the other samples within that plot. Lastly, to test the effect of Day × Treatment, we fit an adonis2() model testing the effect of Plot + Day + Day × Treatment, again randomizing the plots freely within blocks, but not within plots. Dispersion was calculated with the function betadisper(). These results were visualized with a dbRDA showing the effect of the interaction of Treatment and Day on composition, conditioning by block with the capscale() function. Centroids and standard error were calculated from the extracted CAP values and plotted in ggplot2.

To assess resistance and resilience, we examined day‐to‐day change in composition and abundance with several methods. Firstly, pairwise PERMANOVA identified significant compositional change between Day 0 and each subsequent day by treatment. With this method, we assessed resistance by how long the communities resisted significant compositional change following the salinity disturbance, and resilience by how quickly the community returned to a pre‐disturbance community composition (not significantly different from Day 0). Due to the difference in composition found between treatments on Day 0 (treatment effect, see Section 3), we compared daily composition to the Day 0 composition of each respective treatment, instead of to the control. This method identifies how each treatment deviates from its initial community, which more accurately describes community changes than comparing the treatments to the control since their initial communities differed (Table S2 for daily compositional comparisons of each treatment to the control). After first subsetting the data by treatment, then by day (so that each dataframe contained only two time points, Day 0 and one other day), we used the adonis2() function with the how() function as described above to account for repeated measures (permuting samples within plots, but not permuting plots freely). Resistance was assessed based on if or how quickly community composition significantly changed from Day 0. Resilience was assessed by if or how quickly the community returned to a composition similar to the Day 0 composition. To visualize the results, we plotted the effect of Day on community composition with a dbRDA conditioned on block for each treatment. The treatments were ordinated separately to more accurately see how the bacterial composition changes from Day 0 in each treatment using the function capscale() conditioned on block. Spider plots show the centroids per day, calculated based on extracted CAP values, connected to each individual sample point, plotted in ggplot2.

In addition, we considered resistance in terms of the degree of community change following the disturbance by using Bray Curtis dissimilarity. We quantified the Bray Curtis dissimilarity between the Day 0 community of each treatment and every subsequent day. Higher values indicate more compositional change, repressing lower resistance. We also compared the Bray Curtis Dissimilarity between Day 0 and the day that each treatment underwent significant composition change in response to the salinity addition (Day 1 (novel) and Day 3 (repeated), see Section 3). This allows us to compare the degree of change that each treatment experienced and identify with treatment underwent more extreme change. We used the function beta.pair.abund() from the R package betapart (Baselga et al., 2023) to create a dissimilarity matrix. We extracted the dissimilarity values between the Day 0 and every subsequent day per plot to compare dissimilarity between the treatments. Using the same linear model as described for the salinity tests, we compared how dissimilarity from Day 0 varied by treatment, and the same post hoc method as described above was used to assess significance between days and treatments.

To assess how abundance of key taxa changed over time and between treatment, we used a similarity percentage analysis with the function simper() in the R packaged vegan. This analysis calculates the average contribution of each taxon to the community dissimilarity between sample units. Permutations then calculate if the contribution to dissimilarity is significant per taxa. We considered the dissimilarity between the three treatments (control‐repeated disturbance, control‐novel disturbance, repeated disturbance‐novel disturbance). Using unrarefied, relative abundance data, we identified the 100 ASVs that most significant contributed to dissimilarity between each treatment comparison (300 total). Some of the 300 ASVs were present in more than one comparison, so after repeats were removed, there were 254 ASVs (the repeated taxa were still present in the analysis, but only listed once, resulting in a total of 254). To visualize abundance changes in these taxa over time, we subset our data to only include these 254 ASVs. Abundance values were log transformed and plotted as a heatmap using the function pheatmap() in the R package pheatmap (Kolde, 2019), with abundance values cantered and scaled and taxa summed and labelled by the phylum. All statistics and figures were run in R 4.1.2 (R Core Team, 2023).

## RESULTS

### Salinity disturbance

**FIGURE 1:**
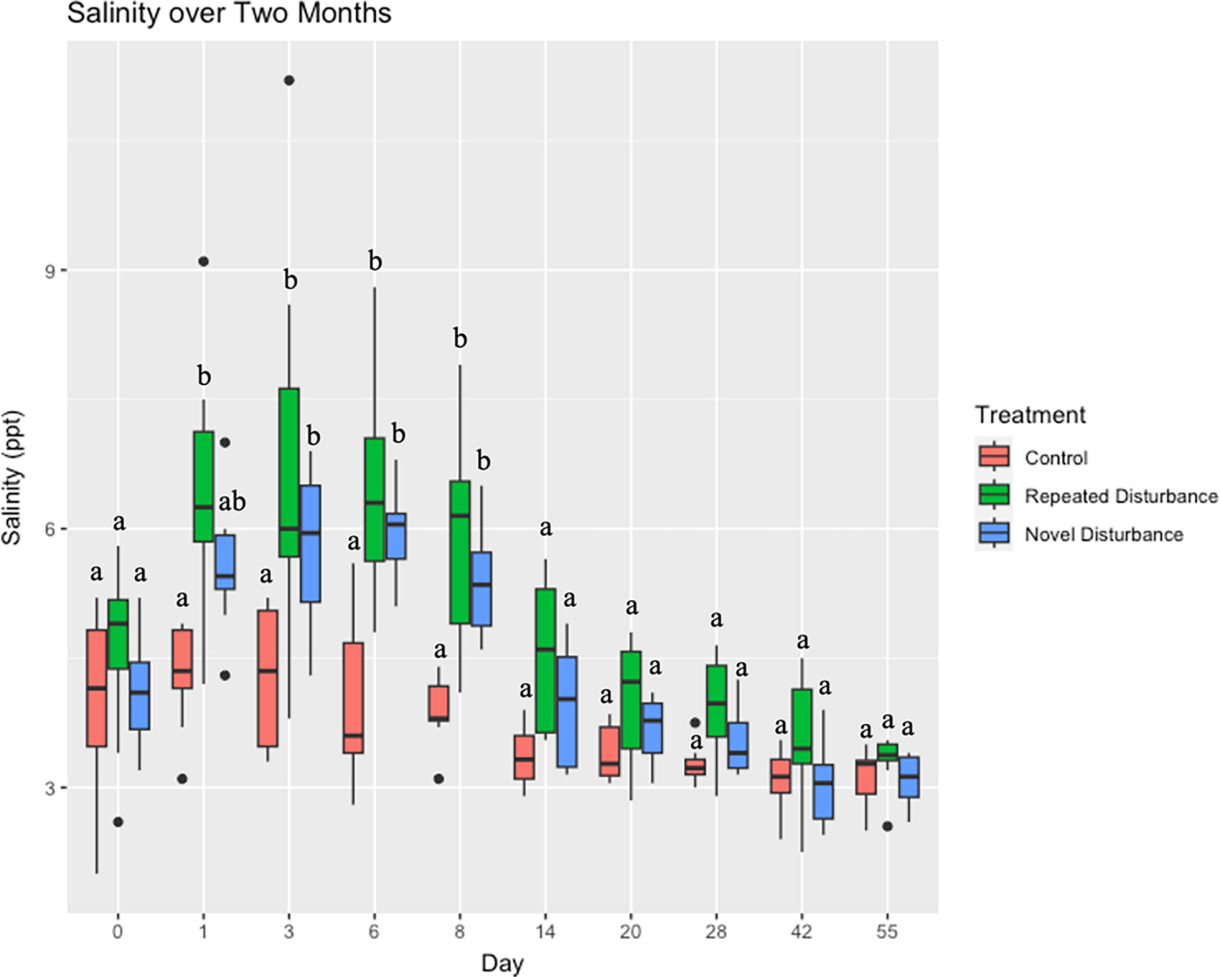
A boxplot of the salinity of each treatment over the two‐month sample collection. Salt was added after salinity was measured on Day 0. Changes in the control represent ambient salinity changes in the system. Significant differences (*p* < 0.05) in salinity per day between treatments are based on post‐hoc tests (Table S1).

The salinity addition significantly increased salinity in the treatment plots for 8 days, and by Day 14 salinity returned to pre‐treatment levels (Figure 1, Table S1). We consider the disturbance phase to last from Day 1 through Day 8, and the recovery phase to begin on Day 14. This timeline of salinity elevation is consistent with salinity measurements taken during the 2‐year disturbance treatment to confirm the effect of the repeated salt additions, which showed salinity returning to ambient conditions after about 2 weeks.

### Effect of repeated salinity additions on community composition

**FIGURE 2:**
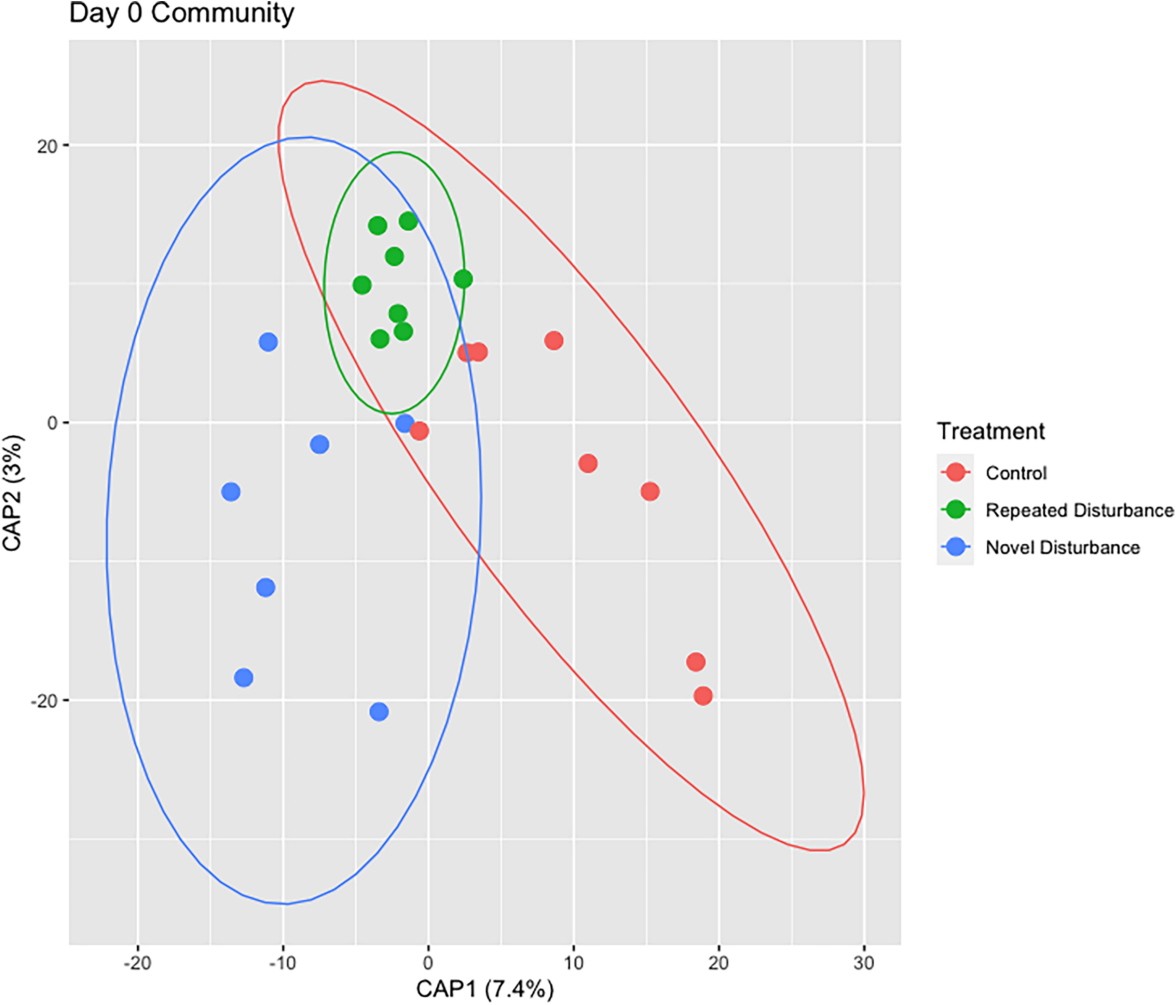
dbRDA plotting the effect of Treatment, conditioned by block, on community composition on Day 0.

The Day 0 community composition significantly differed between treatments (Figure 2; *R* ^2^ = 0.116, pseudo *F*_(2,20)_ = 1.31, *p* = 0.014), and pairwise PERMANOVAs comparing treatments find that the repeated disturbance composition was significantly different from the control (*R* ^2^ = 0.088, pseudo *F*_(1,14)_ = 1.34, *p* = 0.039) and the novel disturbance (*R* ^2^ = 0.087, pseudo *F*_(1,13)_ = 1.23, *p* = 0.039), but the novel disturbance and control did not differ (*R* ^2^ = 0.093, pseudo *F*_(1,13)_ = 1.33, *p* = 0.094). There was no significant difference in dispersion (compositional variance) between treatments (*F* = 1.98, *p* = 0.163), however, the repeated disturbance treatment showed a non‐significant trend of decreased variance compared to the other treatments.

### Effect of repeated salinity additions on disturbance response

**FIGURE 3:**
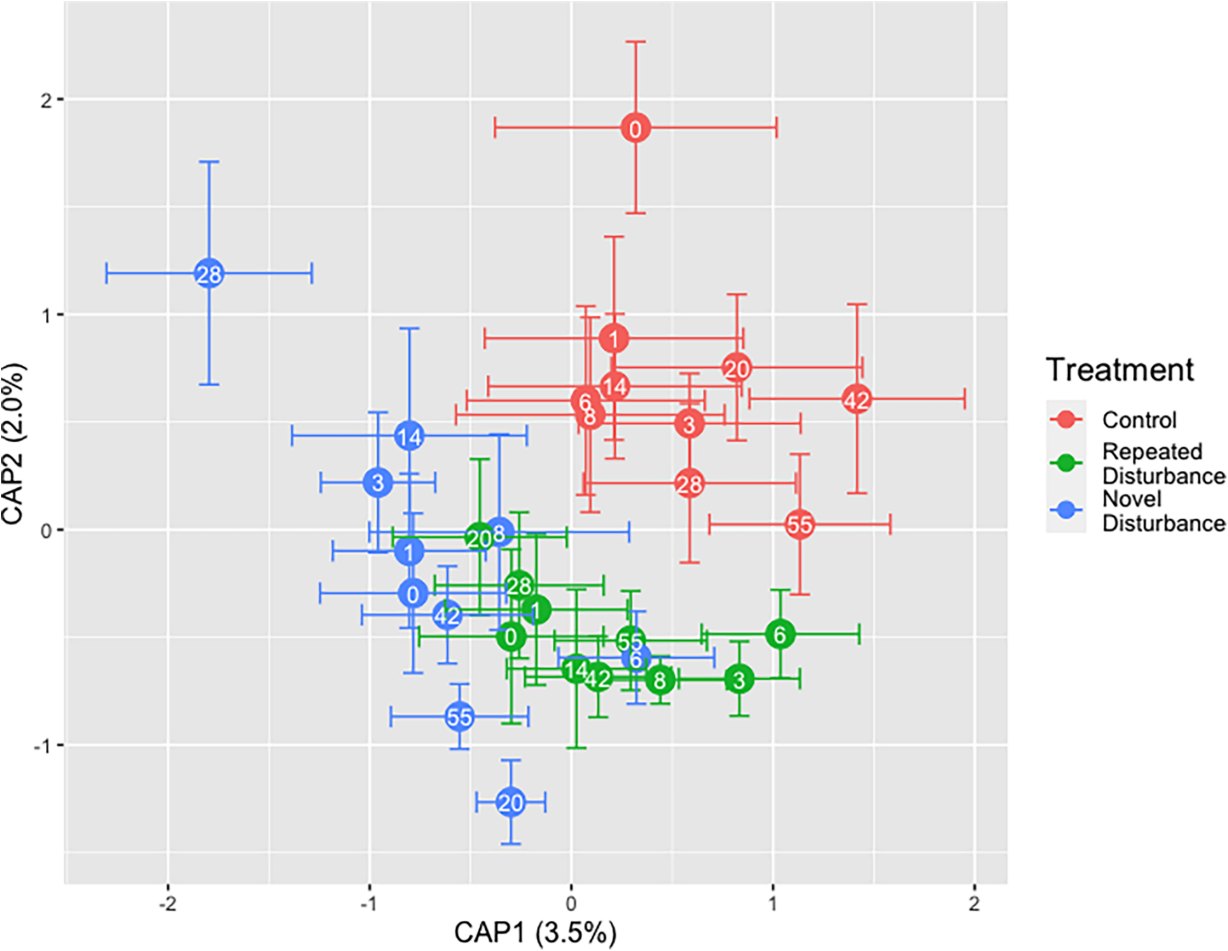
dbRDA plotting the effect of the Treatment × Day interaction, conditioned by block, on community composition. Points represent the centroid of community composition on a given day by Treatment and bars represent standard error. Centroids are labelled by day.

Over the 2 months following the salinity addition, community composition significantly differed based on the disturbance regime (Treatment effect; pseudo *F*_(2, 177)_ = 2.05, *p* = 0.038) and days since disturbance (Day effect; pseudo *F*_(9, 153)_ = 1.67, *p* = 0.001), and the disturbance communities underwent different recovery trajectories over time (Treatment × Day interaction; pseudo *F*_(18, 135)_ = 1.19, *p* = 0.0013) (Figure 3). Over the whole experiment, dispersion was significantly different by Treatment (*F* = 6.00, *p* = 0.003) and by Day (*F* = 2.16, *p* = 0.027). Like the Day 0 trend, the repeated disturbance treatment had lower compositional variance than the other treatments (Figure S1). These results support our second hypothesis, that the treatment communities respond to the disturbance differently based on their past disturbance regime.

### Resistance

**FIGURE 4:**
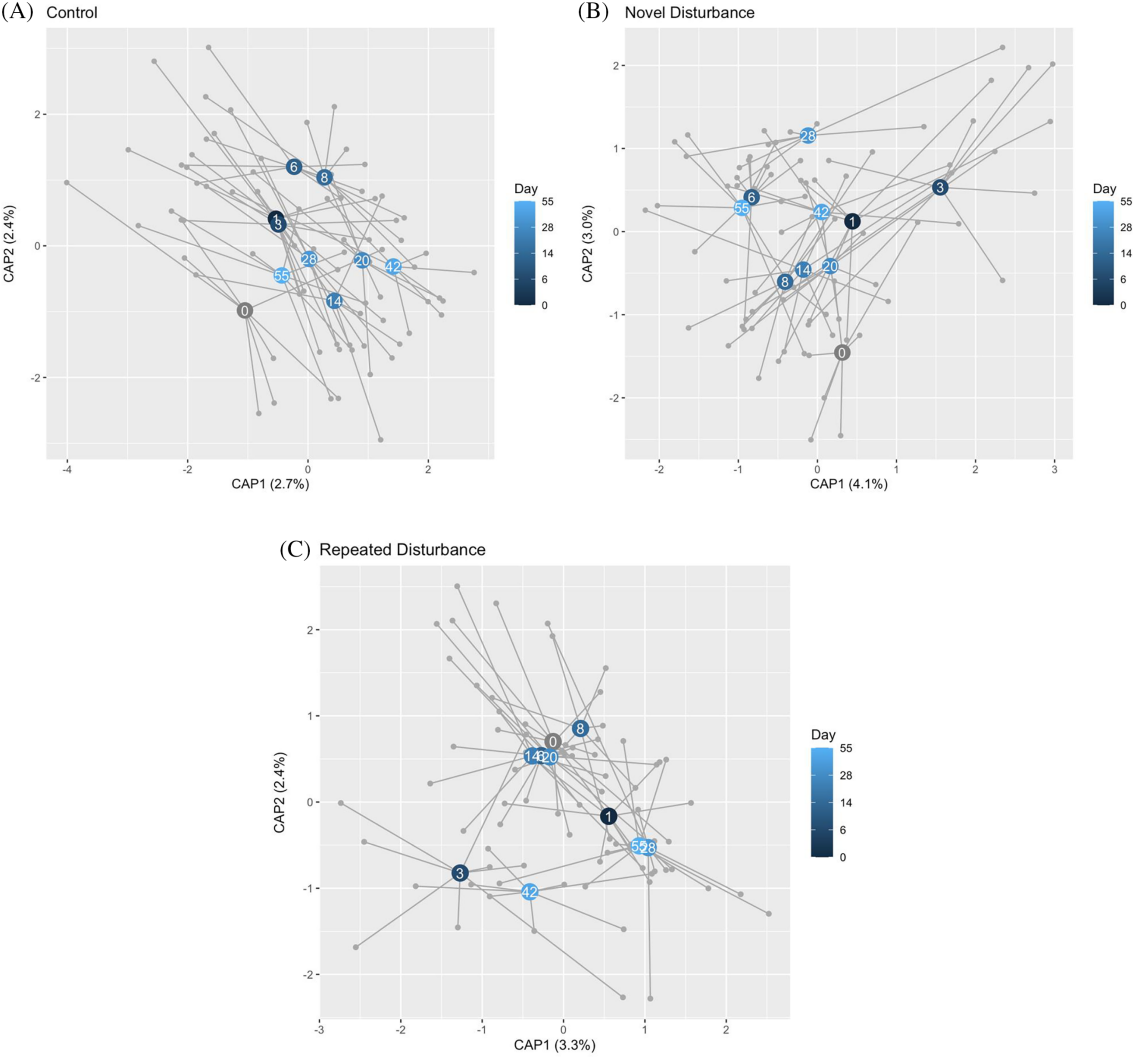
(A–C) dbRDAs plotting the community composition of each day by treatment (ordinated separately): (A) control, (B) novel disturbance, (C) repeated disturbance. Centroids of each day at labelled, and segments show the distance of each individual points (grey points) from the daily centroid.

We found a slight increase in resistance in the repeated disturbance community compared to the novel disturbance based on how quickly the communities underwent significant compositional change following the salinity disturbance (Table 1, Figure 4A–C). The repeated disturbance treatment had only one day of significant compositional change away from the starting community, on Day 3, and the variance of community composition never changed. The novel disturbance underwent multiple days of compositional change, including on Day 1. This indicates lower resistance and a rapid response to the salinity disturbance. The novel disturbance also showed the only significant change in compositional variance, which significantly decreased on Day 6 compared to Day 0. Overall, the control had multiple days of significant compositional change, demonstrating ambient bacterial dynamics.

**TABLE 1:** Results of pairwise PERMANOVAs comparing the composition of each treatment on Day 0 to every subsequent day.

Day comparison	Repeated disturbance		Novel disturbance		Control	
	*p*‐value	Dispersion	*p*‐value	Dispersion	*p*‐value	Dispersion
Day 0–1	0.64	0.64	0.0078**	0.24	0.063^†^	0.46
Day 0–3	0.015*	0.72	0.63	0.25	0.19	0.46
Day 0–6	0.70	0.67	0.38	0.040*	0.016*	0.30
Day 0–8	0.40	0.98	0.45	0.40	0.063^†^	0.26
Day 0–14	0.87	0.80	0.57	0.82	0.14	0.70
Day 0–20	0.90	0.64	0.46	0.95	0.0078**	0.23
Day 0–28	0.98	0.43	0.0078**	0.90	0.070^†^	0.19
Day 0–42	0.94	0.41	0.13	0.47	0.078^†^	0.60
Day 0–55	0.996	0.40	0.047*	0.724	0.063^†^	0.43

*Note*: Significance is represented as follow: ^†^ *p* < 0.1, *p < 0.05, **p < 0.01.

**FIGURE 5:**
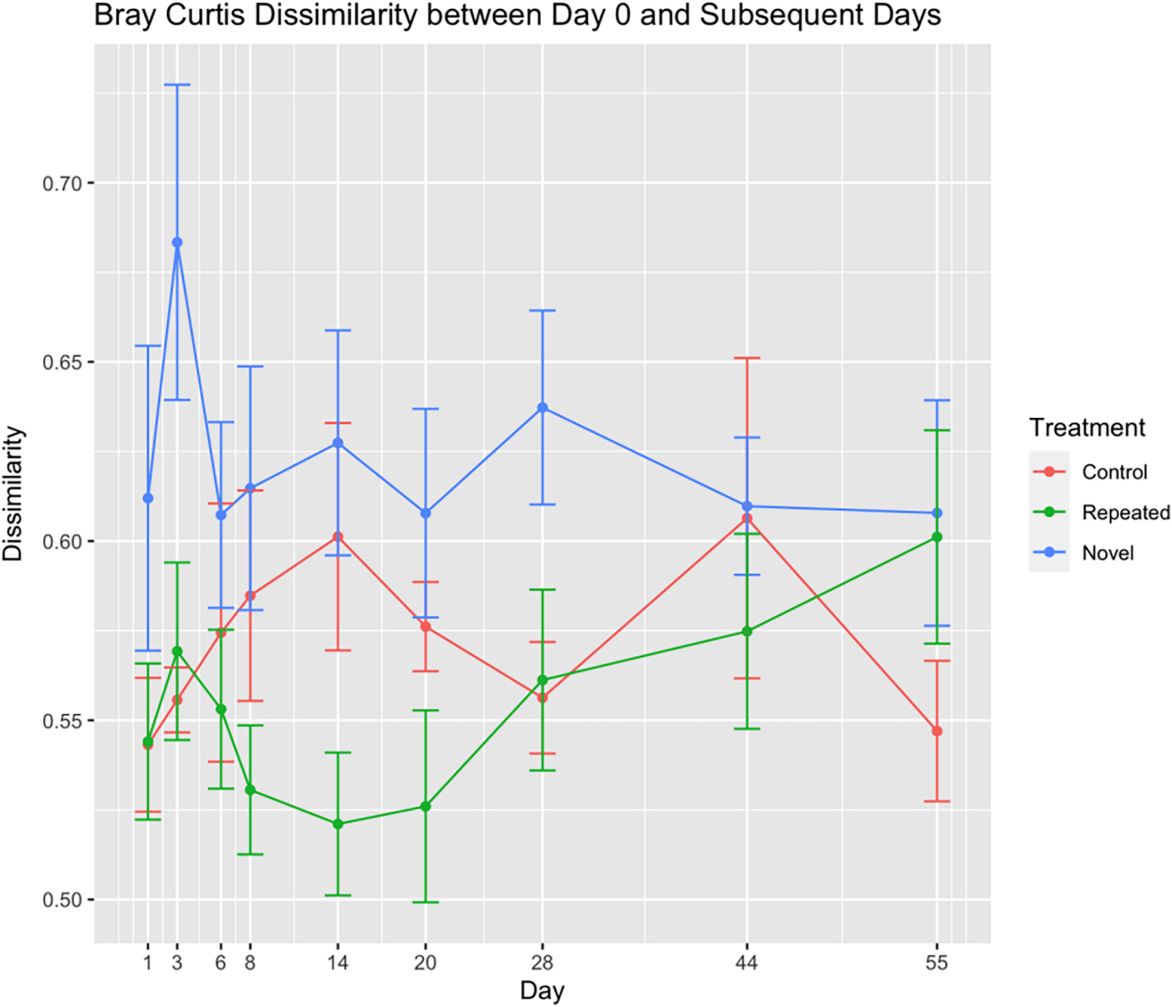
Bray Curtis dissimilarity between the Day 0 community and each subsequent day by treatment. The novel disturbance had significantly higher dissimilarity than the control (*p* = 0.005) and repeated disturbance (*p* = 0.023) on Day 3. On Day 14, the novel disturbance dissimilarity was significantly higher than the repeated disturbance (*p* = 0.047), but not different from the control.

We also used Bray Curtis Dissimilarity to assess resistance by quantifying the degree of community change on each day compared to Day 0. Dissimilarity over time differed by treatment (df = 13, *F* = 12.77, *p* = 0.0009; Figure 5) and the novel disturbance had higher dissimilarity over the sampling period than the repeated disturbance and the control, supporting our prediction. Post hoc tests show that dissimilarity on Day 3 was significantly higher in the novel disturbance community than the repeated disturbance (*p* = 0.012) and the control (*p* = 0.003). We also assessed the degree of change by comparing the dissimilarity of both salt treatments on the day that they underwent significant composition change based on the PERMANOVA results (novel: Day 1, repeated: Day 3). While the novel disturbance had higher dissimilarity, the difference was not significant (*p* = 0.89). Together with the PERMANOVA result, we found a moderate increase in resistance in the repeated disturbance treatment compared to the novel disturbance.

### Resilience

We found equally high resilience in both treatments based on how quickly community composition recovered after undergoing significant compositional change. Both salt treatments experienced one day of significant composition change during the disturbance phase, and both immediately returned to a composition similar to Day 0, even while salinity was still elevated. This indicates high resilience in both communities. After salinity returned to normal (Day 14 and beyond) the community composition and abundance continued to change in the control and novel disturbance but remained constant in the repeated disturbance.

**FIGURE 6:**
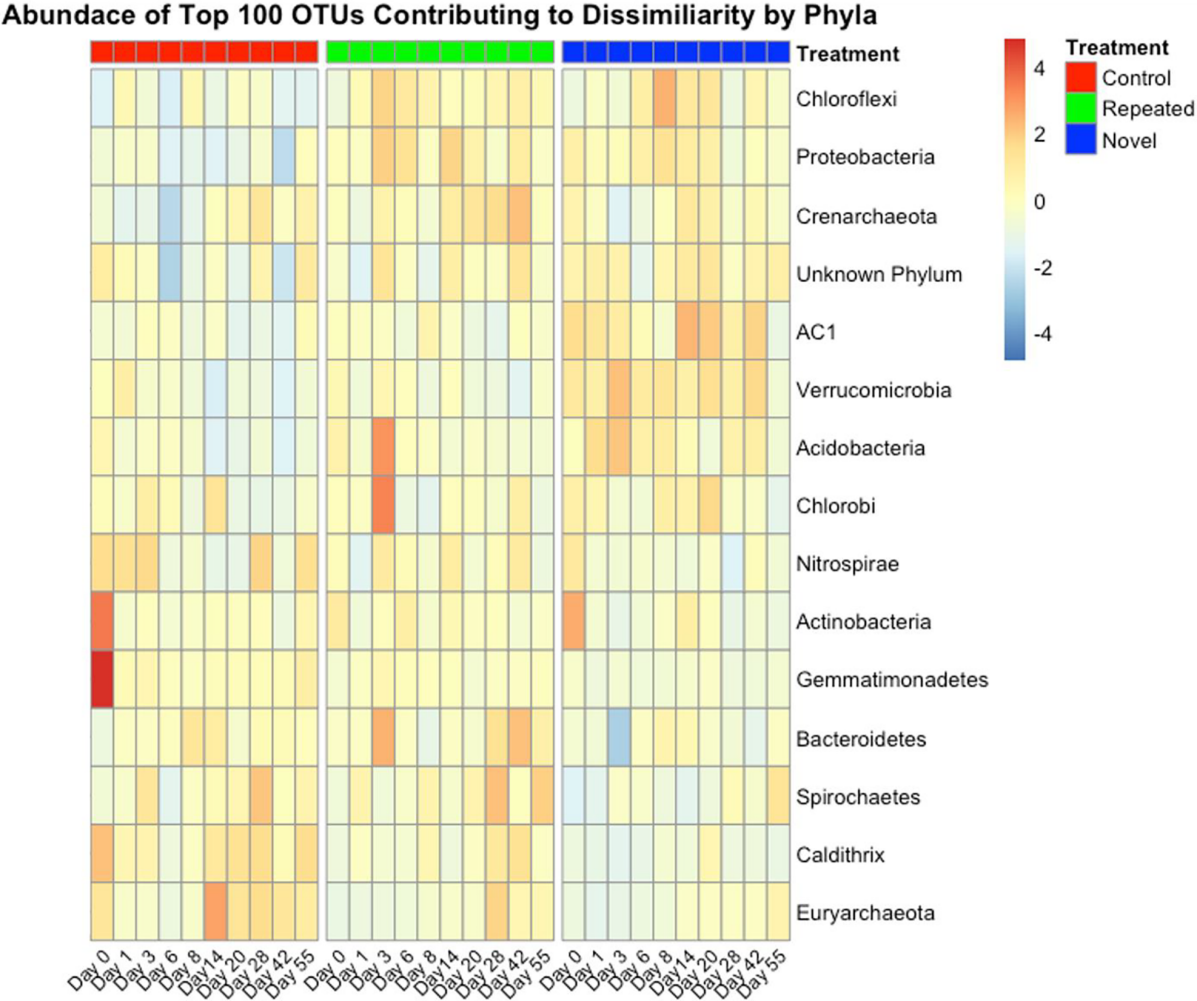
Heatmap plotting the abundance of the 100 taxa that significantly contributed the most to community dissimilarity by treatment (total 254), labelled by phylum. Warm colours represent high abundance and cool colours represent low abundance. The columns are arranged first by treatment, constituting each panel and indicated with colours on the top of the figure. Within each panel, the columns are in chronological order by day, labelled on the bottom of the figure. Full taxonomic classification of the ASVs represented in this analysis and figure can be found in Table S3.

To further examine resilience, we used a heatmap to plot relative abundance changes of the taxa that most significantly contributed to community dissimilarity between treatments based on a similarity percentage analysis (Figure 6, Table S3 for full taxonomy). In the control, Actinobacteria, Gemmatimonadetes, Caldithrix, Nitrospirae, and Euryarchaeota were among the most abundant phyla. The repeated disturbance had high abundance of Acidobacteria, Chlorobi, Chloroflexi, Proteobacteria, and Bacteroidetes. The novel disturbance was dominated by Actinobacteria, Acidobacteria, Verrucomicrobia, Chloroflexi, and AC1 (a phylum in Greengenes).

There were notable changes in abundant taxa in the salt treatments before and after the salinity addition and over the course of the experiment. On Day 0, the novel disturbance treatment was like the control with high abundance of Actinobacteria. However, immediately following the salinity addition (Day 1), the abundance of Actinobacteria decreased in the novel disturbance, suggesting the salt sensitivity of this phylum. Interestingly, several phyla increased in abundance following the salinity addition, but differed in their abundance patterns between salt treatments. Acidobacteria was amplified in both treatments following the addition, but immediate recovered in the repeated disturbance while remaining high in the novel disturbance. Chlorobi and Bacteroidetes increased in the repeated treatment only, while the latter decreased in the novel treatment. Verrucomicrobia and AC1 were only elevated in the novel disturbance treatment.

While there were many abundance changes unique to each salt treatment, overall, there was a pattern of phyla abundance spiking and quickly recovering in the repeated disturbance compared to abundance increases that were maintained in the novel disturbance. By the final timepoints, the repeated disturbance is similar to the control, with elevated Spirochaetes, Caldithrix, and Euryarchaeota, suggesting recovery, while the novel disturbance did not show taxonomic recovery.

## DISCUSSION

This study examined the effect of past disturbances on soil bacterial composition and disturbance response. We hypothesized that communities with a salinity disturbance history will differ from those that have not experienced an experimental disturbance, and that their recovery from a subsequent salinity disturbance will differ. Overall, we found support for both hypotheses.

This experiment detected bacterial community compositional changes within days following an environmental disturbance. This rapid timescale is consistent with other lab and mesocosm research (Ager et al., 2010; Berga et al., 2012; Hu et al., 2018; Jurburg, Nunes, Brejnrod, et al., 2017; Rodríguez‐Valdecantos et al., 2017; Shade et al., 2011) and is the fastest timescale of microbial community change found in nature as far as we are aware. The control treatment captured the ambient bacterial dynamics that occur across 2 months, demonstrating how variable communities can be over time. This result helps inform our understanding of natural soil temporal dynamics in wetlands.

### Effect of long‐term disturbances on composition

The repeated salinity disturbances over 2 years altered community composition, as Day 0 composition differed between the repeated disturbance treatment and the treatments that had not experience past disturbances (novel, control). While the methods used do not identify a mechanism, this points to the salinity addition selecting for salt tolerant taxa. Salinity is an important factor in structuring bacterial communities (Lozupone & Knight, 2007), and community salt tolerance has been found to be proportional to soil salinity (Rath et al., 2019). Our results show that the monthly salinity addition, which increased salinity by ~33% for about 2 weeks, or half of the time for 2 years, constituted a significant disturbance to the ambient salinity regime and cultivated a bacterial community adapted to altered salinity.

### Recovery trajectories

We found that the repeated and novel disturbance treatments underwent different recovery trajectories following the salinity disturbance, supporting our second hypothesis. The differences in their trajectories were seen in compositional differences over the recovery period (interactive effect), elevated relative abundances of distinct taxa, and differences in community variance. While both salt treatments had similar responses to the salinity disturbance, the relative abundance results show the repeated disturbance recovered taxonomically while the novel disturbance does not, which could reflect the compositional results. The treatments also differed in terms of community variance, where the repeated disturbance had consistently lower variance than the other treatments, and the novel disturbance had a sharp decrease in dispersion following the salinity disturbance but recovered after the first week. This suggests that the salinity disturbance decreases community variance, likely due to the death of salt sensitive taxa (Wichern et al., 2006). The low variance in the repeated disturbance treatment, both on Day 0 and following the salinity addition, suggest that the past disturbances had a strong filtering effect on the community.

### Disturbance response: resistance

The repeated disturbance treatment increased community resistance to subsequent disturbances, as we predicted, but only slightly. The salinity addition led to compositional changes in the novel disturbance community on Day 1, and the repeated disturbance community changed on Day 3. While this result demonstrates increased resistance, as has been found in other repeated disturbance studies (Bérard et al., 2012; Bouskill et al., 2013; Canarini et al., 2021), the difference between the treatments was only one sampling time point, representing only a modest increase.

We also considered resistance in terms of degree of community change using dissimilarity, which also demonstrated a modest increase in resistance in the repeated disturbance treatment. During the disturbance phase, the novel disturbance community had higher dissimilarity than the repeated disturbance, indicating more extreme community changes. This generally supports our prediction, but with one notable exception. We anticipated that the novel disturbance would undergo more extreme compositional change than the repeated disturbance during its initial disturbance response (Day 1 and Day 3, respectively). However, we did not find a difference in dissimilarity between the novel treatment on Day 1 and the repeated treatment on Day 3, suggesting they both underwent similar degrees of change in the immediate response to salinity. Taken together, the resistance results show that (1) the initial community response to the salinity disturbance was slightly delayed in the repeated disturbance treatment due to past exposures, (2) the salt treatments underwent the same degree of community change in response to the initial disturbance, and (3) the repeated disturbance community remained more like its pre‐treatment type over the disturbance phase than the novel disturbance.

The mechanisms that caused the slight increase in resistance are unknown. The repeated salinity additions could have filtered out salt sensitive taxa (Logares et al., 2013; Rath et al., 2019) as the decrease in community variance in the repeated disturbance treatment would suggest. The past disturbances could also have selected for taxa with an improved ability to withstand stressful conditions through adaptations like increased dormancy potential (Kearns & Shade, 2018). While our methods removed relic DNA to capture a clearer signal of community change, they did not differentiate between the active and dormant community. If certain taxa adapted to survive frequent salinity pulses by increase dormancy potential, they would still be detected in our sampling and result in fewer compositional changes. Barnett and Shade (2024) compared the resilience of the whole bacterial community to only the active (non‐dormant) community by comparing DNA and RNA sequencing and found stronger recovery patterns in the whole community than the active subset. This suggest that dormancy and the microbial seedbank are critical for community disturbance response and might explain our results.

Other studies of disturbance dynamics have found that disturbances select for microbial specialists (Renes et al., 2020) and tolerant taxa (Jurburg, Nunes, Stegen, et al., 2017), or cause bacteria to adopt new life strategies to withstand disturbances (Evans & Wallenstein, 2014). Through evolution and/or horizontal gene transfer, these traits could have increased resistance to future salinity disturbances. Bacteria have been found to evolve stress tolerance in 250–2000 generations (Zhou et al., 2017), which is within the timeframe of the two‐year repeated disturbance conditioning phase and could explain our results. These adaptations would lead to increased community resistance to a repeated disturbance, but more research is needed to understand which mechanisms are more important in driving microbial compositional changes in nature.

### Disturbance response: resilience

Overall, we found resilience in this system in both the repeated and novel disturbance treatments, but the heat map suggest higher resilience in the repeated disturbance community, as expected. While the rapid community response to the salinity addition during the disturbance phase was notable, perhaps more surprising was the immediate recovery in both salt‐disturbed treatments. We predicted that both communities would exhibit high resilience due to the frequent abiotic fluctuations in the system, but we did not expect recovery to happen while salinity was still elevated. Other studies have found bacterial communities to recover from a disturbance in about 25 days (Jurburg, Nunes, Brejnrod, et al., 2017), but more work examining bacterial community changes over short time periods would be beneficial to understand community recovery patterns on this time scale. Our results show that the repeated disturbance community maintained its post‐recovery community (Day 6) for the remainder of the experiment, while the novel disturbance and control communities continued to shift over time. This, along with the decreased community variance, suggests that the past salinity additions had a strong filtering effect on the taxa present and continues to impact the community dynamics beyond the recovery phase.

The focus of this study was on compositional responses to disturbance, but there were notable changes in the abundances of phyla known to be salt sensitive/tolerant and known as either nitrogen or sulphur cyclers, suggesting potential functional differences between treatments. Firstly, the control had high abundances of salt‐sensitive phyla, such as Actinobacteria and Gemmatimonadetes (Li et al., 2021; Wijaya et al., 2022), and the repeated disturbance was defined by high abundance of salt‐tolerance taxa, like Bacteroidetes and Proteobacteria (Mhete et al., 2020; Wijaya et al., 2022). The control had higher abundance of phyla know as nitrogen cyclers, like Nitrospira (Chen et al., 2022; Mhete et al., 2020), while the salt treatments had high abundance of sulphur cycling phyla, like Chlorobi (Jagannathan & Golbeck, 2009; Kuypers et al., 2018) and Proteobacteria (Arora, 2017; Wasmund et al., 2017). These results support other research finding that nitrogen fixers and nitrogen cycling genes decrease as soil salinity increases (Li et al., 2021; Morrissey & Franklin, 2015) while Proteobacteria (particularly sulphur‐reducing classes) increase in abundance with salinity (Li et al., 2021, Morrissey & Franklin, 2015). Microbial communities are often considered to have high functional redundancy, but recent studies have found recovery patterns are decoupled between composition/diversity and soil community function, demonstrating the importance of considering the resilience of both community structure and function (Choi et al., 2017; Sjöstedt et al., 2018). It is possible that the repeated salinity disturbances in our experiment could have cultivated a community with different functions and altered nutrient availability, but a focussed examination of microbial function would be necessary to determine this.

### Limitations

The central limitations of this study are rooted in the challenges of field‐based microbiome surveying. Soil collection required destructive sampling, so the same location and, potentially, community could not be repeatedly sampled within our plots. Instead, samples were collected over time from randomly chosen sub‐plots. Our methods attempted to account for this by distributing salt across the plot as evenly as possible, measuring salinity from multiple plot locations, and taking care to ensure all plots had a similar and homogenous plant community; however, samples were collected from a new location in the plot on each sampling day which therefore introduced unknown community variance. The effects of the interacting plant community were also not considered, though care was taken to ensure all plots had a similar plant community and that collection was done outside of growing season to reduce plant effects on the soil microbes. The molecular methods used do not distinguish between active and dormant bacteria and do not focus on functional differences between treatments. Further investigation of these specific areas would provide greater insight into the mechanisms microbes utilized to withstand disturbance and functional consequences of disturbance events.

## CONCLUSION

In conclusion, this study found long‐term, past disturbances to alter bacterial community composition and response to future disturbances. We identified moderate increases in resistance and resilience to disturbance based on the community's exposure to past disturbances, supporting similar results found in systems with different disturbances, mainly drought. Furthermore, we found soil bacterial to undergo significant compositional change following a salinity disturbance in a matter of days, confirming the short timescale of bacteria turnover found in lab‐based experiments. These results suggest that soil microbiomes are likely well‐adapted to typical abiotic fluctuations and are resilient to disturbances, but novel disturbances may alter community structure and function.

## AUTHOR CONTRIBUTIONS

**Susannah Halbrook:** Conceptualization; writing – original draft; methodology; formal analysis. **William Wilber:** Conceptualization. **Mary Elizabeth Barrow:** Methodology. **Emily C. Farrer:** Funding acquisition; writing – review and editing; formal analysis.

## CONFLICT OF INTEREST STATEMENT

The authors declare no conflict of interest.

## Supporting information


**TABLE S1:** Post‐hoc test results comparing salinity between the three treatments on each day. Significance is represented as follow: ^†^ *p* < 0.1, *p < 0.05, **p < 0.01, ***p < 0.001.
**FIGURE S1:** Dispersion values for the three treatments on each day were extracted from the R object produced from calculated dispersion with the function betadisper() in the R package Vegan. The dispersion values per day were extracted from $group.distances, which results in one value per treatment per day, and therefore there are no means or quartiles plotted.
**TABLE S2:** Pairwise PERMANOVAs comparing the composition on each day between treatments. PERMANOVAs were done by subsetting the data first by Day, and then by the two treatments to be compared. This method did not require manual PERMANOVAs because there were no repeated measures. The function adonis2() was used for the argument strata conditioning on the block. Dispersion was calculated with the function betadisper().

**TABLE S3:** Full taxonomy of the taxa identified as the top 100 taxa most significantly contributing to the dissimilarity between treatments based on a similarity percentage analysis. The top 100 taxa were identified per treatment comparison.

## Data Availability

The data that support the findings of this study are openly available in NCBI GenBank Sequence Read Archives (SRA), reference number PRJNA1105352 https://www.ncbi.nlm.nih.gov/bioproject/PRJNA1105352.
