# Supplementary material for: Bacterial community response to novel and repeated disturbances
Source: Environ Microbiol Rep. 2024 Oct 10;16(5):e70022. doi: 10.1111/1758-2229.70022 (PMC11465558; doi:10.1111/1758-2229.70022)
Supplement: Supplementary file 1 — TABLE S1: Post‐hoc test results comparing salinity between the three treatments on each day. Significance is represented as follow: † p < 0.1, *p < 0.05, **p < 0.01, ***p < 0.001. FIGURE S1: Dispersion values for the three treatments on each day were extracted from the R object produced from calculated dispersion with the function betadisper() in the R package Vegan. The dispersion values per day were extracted from $group.distances, which results in one value per treatment per day, and therefore there are no means or quartiles plotted. TABLE S2: Pairwise PERMANOVAs comparing the composition on each day between treatments. PERMANOVAs were done by subsetting the data first by Day, and then by the two treatments to be compared. This method did not require manual PERMANOVAs because there were no repeated measures. The function adonis2() was used for the argument strata conditioning on the block. Dispersion was calculated with the function betadisper(). [file EMI4-16-e70022-s002.docx]

**Supplemental Information and Figures**

**Supplemental Table 1**: Post-hoc test results comparing salinity between the three treatments on each day. Significance is represented as follow: † P<0.1, * P<0.05, ** P<0.01, ***P<0.001

| Day | Treatment Comparison | Z- value | P- value |
| --- | --- | --- | --- |
| Day 0 | Repeated vs Control | 1.41 | 0.96 |
|  | Novel vs Control | 0.24 | 1.00 |
|  | Repeated vs Novel | 1.17 | 0.99 |
| Day 1 | Repeated vs Control | 5.21 | <0.001*** |
|  | Novel vs Control | 3.04 | 0.06^†^ |
|  | Repeated vs Novel | 2.17 | 0.48 |
| Day 3 | Repeated vs Control | 5.99 | <0.001*** |
|  | Novel vs Control | 4.39 | 0.013* |
|  | Repeated vs Novel | 2.50 | 0.25 |
| Day 6 | Repeated vs Control | 6.08 | <0.001*** |
|  | Novel vs Control | 4.69 | <0.001*** |
|  | Repeated vs Novel | 1.38 | 0.97 |
| Day 8 | Repeated vs Control | 4.93 | <0.001*** |
|  | Novel vs Control | 3.64 | 0.0077** |
|  | Repeated vs Novel | 1.29 | 0.98 |
| Day 14 | Repeated vs Control | 2.86 | 0.10 |
|  | Novel vs Control | 1.46 | 0.95 |
|  | Repeated vs Novel | 1.40 | 0.907 |
| Day 20 | Repeated vs Control | 1.44 | 0.95 |
|  | Novel vs Control | 0.69 | 1.00 |
|  | Repeated vs Novel | 0.75 | 1.00 |
| Day 28 | Repeated vs Control | 1.62 | 0.88 |
|  | Novel vs Control | 0.63 | 1.00 |
|  | Repeated vs Novel | 0.99 | 1.00 |
| Day 42 | Repeated vs Control | 1.19 | 0.99 |
|  | Novel vs Control | -0.09 | 1.00 |
|  | Repeated vs Novel | 1.28 | 0.99 |
| Day 55 | Repeated vs Control | 0.42 | 1.00 |
|  | Novel vs Control | -0.09 | 1.00 |
|  | Repeated vs Novel | 0.51 | 1.00 |

**Supplemental Figure 1:** Dispersion values for the three treatments on each day were extracted from the R object produced from calculated dispersion with the function betadisper() in the R package Vegan. The dispersion values per day were extracted from $group.distances, which results in one value per treatment per day, and therefore there are no means or quartiles plotted.


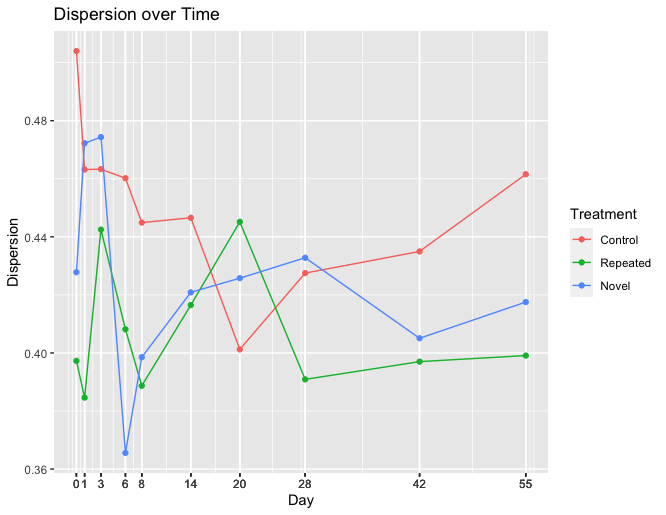


**Daily pairwise treatment comparisons**

To analyze resistance by treatment, we compared the daily community composition to the Day 0 composition of each treatment separately since the Day 0 communities were different between treatments. This method focuses on how each treatment deviates from its initial community. However, we also compared the daily community composition between treatments, which is useful for looking at overall patterns in compositional differences (Supplement Table 2). We find that the repeated disturbance treatment has multiple days of significant compositional difference from the control, while the novel disturbance does not differ (two days with non-significant trends, p=0.055). Similarly, the repeated and novel disturbance treatments only significantly differed on Day 1, before the novel disturbance received salt (Figure 2), and then only exhibited a few days of differences that were non-significant. This indicates that, overall, the largest difference between treatment communities is between the repeated disturbance and the control, due to past salinity disturbances. The novel disturbance community appears to be somewhat intermediate between the repeated disturbance and the control due to compositional changes following the salinity addition. Again, we choose to only view these results generally, as the initial differences between the treatment composition suggest that we may not expect the communities of each day to be similar, so comparing them is not the most accurate way of assessing resistance.

**Supplemental Table 2:** Pairwise PERMANOVAs comparing the composition on each day between treatments. PERMANOVAs were done by subsetting the data first by Day, and then by the two treatments to be compared. This method did not require manual PERMANOVAs because there were no repeated measures. The function adonis2() was used for the argument strata conditioning on the block. Dispersion was calculated with the function betadisper().

| Day | **Repeated-Control** | | **Novel-Control** | | **Repeated-Novel** | |
| --- | --- | --- | --- | --- | --- | --- |
|  | **P value** | **Dispersion** | **P value** | **Dispersion** | **P value** | **Dispersion** |
| Day 0 | 0.031* | 0.068^†^ | 0.094 | 0.096 | 0.031* | 0.76 |
| Day 1 | 0.30 | 0.073^†^ | 0.57 | 0.57 | 0.10 | 0.0045** |
| Day 3 | 0.20 | 0.84 | 0.13 | 0.73 | 0.055^†^ | 0.56 |
| Day 6 | 0.047* | 0.80 | 0.20 | 0.10 | 0.078^†^ | 0.14 |
| Day 8 | 0.22 | 0.42 | 0.15 | 0.82 | 0.45 | 0.46 |
| Day 14 | 0.039* | 0.52 | 0.20 | 0.65 | 0.55 | 0.77 |
| Day 20 | 0.086^†^ | 0.23 | 0.055^†^ | 0.33 | 0.60 | 0.53 |
| Day 28 | 0.38 | 0.40 | 0.20 | 0.77 | 0.14 | 0.40 |
| Day 42 | 0.040* | 0.71 | 0.055^†^ | 0.75 | 0.055^†^ | 0.91 |
| Day 55 | 0.18 | 0.17 | 0.16 | 0.60 | 0.48 | 0.42 |

**Supplemental Table 3:** Full taxonomy of the taxa identified as the top 100 taxa most significantly contributing to the dissimilarity between treatments based on a similarity percentage analysis. The top 100 taxa were identified per treatment comparison.
